# HIV drug resistance, early treatment outcomes and impact of guidelines compliance after protease inhibitor‐based second‐line failure in a dedicated resistance clinic in western Kenya: a retrospective cohort study

**DOI:** 10.1002/jia2.26523

**Published:** 2025-06-09

**Authors:** John M. Humphrey, Shamim M. Ali, Allison DeLong, Vlad Novitsky, Edwin Sang, Bilal Jawed, Emmanuel Kemboi, Celia Ngetich, Suzanne Goodrich, Adrian Gardner, Joseph W. Hogan, Rami Kantor

**Affiliations:** ^1^ Department of Medicine Indiana University School of Medicine Indianapolis Indiana USA; ^2^ Department of Medicine Moi University School of Medicine Eldoret Kenya; ^3^ Department of Biostatistics Brown University School of Public Health Providence Rhode Island USA; ^4^ Department of Medicine Brown University Alpert Medical School Providence Rhode Island USA; ^5^ Academic Model Providing Access to Healthcare (AMPATH) Eldoret Kenya; ^6^ Department of Pharmacy Moi Teaching and Referral Hospital Eldoret Kenya

**Keywords:** drug resistance, guideline, HIV, second line, third line, treatment failure

## Abstract

**Introduction:**

Data on drug resistance, viral outcomes and guidelines compliance following protease inhibitor (PI)‐based second‐line failure in low‐ and middle‐income countries are limited, particularly in the era of dolutegravir‐containing antiretroviral therapy (ART).

**Methods:**

We conducted a retrospective cohort study of people living with HIV (PLWH) ≥3 years old with second‐line viral failure (VF, ≥1000 copies/ml) at the Academic Model Providing Access to Healthcare from 2011 to 2021. We assessed resistance prevalence and patterns at second‐line VF, stratified by PI (atazanavir/ritonavir or lopinavir/ritonavir), and examined correlations of resistance and treatment strategies with VF at 6–18 months post‐genotype. Analyses employed inverse probability weighting, adjusting for calendar year, age, gender, ART duration, PI at genotyping and class‐specific resistance, and considered guidelines‐supported versus unsupported strategies.

**Results:**

Of 187 participants (median age 41 years, 54% female, 41% on atazanavir/ritonavir, 59% on lopinavir/ritonavir‐based ART), 91% had any resistance (NRTI 79%, NNRTI 80%, major PI 37%, dual‐class 36%, triple‐class 37%). Predicted resistance to third‐line options was 67% for etravirine or rilpivirine and 10% for darunavir/ritonavir. Despite higher resistance detected on atazanavir/ritonavir versus lopinavir/ritonavir, predicted darunavir/ritonavir resistance was similar. At median 9 months post‐genotype, 95% of 173 participants with available data were on a guidelines‐supported regimen (55% second‐line; 45% third‐line, 86% dolutegravir‐based), of whom 28% had post‐genotype VF. Of the 5% not on guidelines‐supported regimens, 71% had post‐genotype VF. Adjusted odds of VF were higher for guidelines‐unsupported versus supported regimens (OR = 4.52; 95% CI 1.02−26.24), and odds of VF were 97% lower for those on third‐line versus second‐line (OR = 0.07; 95% CI 0.02−0.20).

**Conclusions:**

We found high levels of drug resistance and early VF following PI‐based second‐line failure in Kenya. Treatment guidelines compliance and switches to third‐line, even within guidelines recommendations, improved early viral outcomes. Findings highlight the vulnerability of PLWH with advanced ART experience and resistance profiles, and the importance of following guidelines and improving access to third‐line and drug resistance testing, particularly in the new ART era.

## 1 INTRODUCTION

Global scale‐up of antiretroviral therapy (ART) has increased the number of people living with HIV (PLWH) with failure of protease inhibitor (PI)‐based second‐line following non‐nucleoside reverse transcriptase inhibitor (NNRTI)‐based first‐line failure in low‐ and middle‐income countries (LMICs) [1]. Extensive resistance is reported from some African cohorts, with 20–40% developing viral failure (VF) with multiclass resistance, limiting future ART and survival [2, 3, 4]. These factors underscore the need to optimize third‐line strategies, especially as dolutegravir is integrated into all ART regimens globally [5, 6, 7]. Realities like under‐ or late detection of VF and resistance due to limited monitoring, low compliance with treatment guidelines, and delayed third‐line transitions raise questions about the adequacy and implementation of second‐line guidelines and their real‐world impact [6, 8, 9].

From 2010 to 2016, the World Health Organization (WHO) recommended lopinavir/ritonavir and atazanavir/ritonavir as preferred second‐line PIs after failure of first‐line NNRTI‐based regimens [10, 11, 12]. In Kenya, 79% of PLWH with second‐line failure had resistance, including 57% dual‐ and 7% triple‐class resistance [7]. With an estimated 19% of 65,000 Kenyans on PI‐based second‐line not virally suppressed in 2023, such high resistance and its impact on care remain serious concerns, even in the new ART era [13]. It may indicate adherence challenges or NRTI/NNRTI cross‐resistance, potentially limiting the effectiveness of newer classes, regimens, formulations and drugs, including dolutegravir. WHO introduced dolutegravir‐based ART in 2016 as first‐ or third‐line options, with boosted PIs recommended for PLWH with dolutegravir‐based first‐line failure [12]. By 2023, >90% of countries have adopted dolutegravir as preferred first‐line ART [14]. However, clinic‐level uptake is heterogeneous. Many Kenyans still use lopinavir/ritonavir and atazanavir/ritonavir, keeping them relevant in WHO guidelines and practice, before or after dolutegravir‐based regimens [15].

In Kenya, dolutegravir was first introduced as a third‐line option for adolescents/adults in 2016, and atazanavir/ritonavir was preferred over lopinavir/ritonavir for people ≥15 years due to improved dosing and side effects [16]. Kenya rapidly adopted WHO guidelines, making dolutegravir‐based ART the recommended first‐line in 2018 [17, 18]. Understanding the use of these drugs within evolving guidelines is important to optimizing treatment strategies.

When dolutegravir was incorporated into third‐line WHO and Kenya guidelines, it joined existing third‐line options ritonavir‐boosted darunavir, etravirine and raltegravir [12, 16]. These guidelines were mostly based on data from settings with lower HIV burdens and higher genotyping and third‐line availability than many LMICs [19, 20, 21, 22, 23, 24]. Few studies have characterized outcomes after PI‐based second‐line failure in sub‐Saharan Africa, especially for those with known resistance and since dolutegravir availability [22, 23, 25, 26]. Characterizing resistance patterns upon PI‐based second‐line failure, including differences between atazanavir/ritonavir and lopinavir/ritonavir, and impacts on third‐line ART, including dolutegravir, can inform treatment strategies in LMICs [12, 27, 28].

In this study at a public‐sector HIV drug Resistance Cieselinic in Kenya, we characterized genotypic resistance upon second‐line failure, compared post‐genotype VF among those continuing second‐line versus switching to third‐line, evaluated guidelines compliance and determined predictors of post‐genotype VF. We tested four hypotheses evaluating the impact of guidelines compliance and the extent and specifics of drug resistance upon second‐line failure on treatment outcomes.

## 2 METHODS

### 2.1 Study design and setting

In this retrospective cohort study, we analysed medical records from PLWH at the Academic Model Providing Access to Healthcare (AMPATH) in western Kenya, which provides guidelines‐based HIV services [29]. In 2015, AMPATH established a Drug Resistance Clinic at Moi Teaching and Referral Hospital (MTRH) to provide multidisciplinary, patient‐centred care for those with second‐line failure. The clinic receives referrals from MTRH's general HIV clinics and other AMPATH‐affiliated sites. MTRH serves >15,000 PLWH and AMPATH serves >120,000. Our study included all eligible PLWH referred to the Resistance clinic from MTRH and other AMPATH sites, who underwent genotyping.

During the study, recommended anchor drugs for first‐line regimens included efavirenz or nevirapine for individuals ≥3 years; second‐line regimens included atazanavir/ritonavir or lopinavir/ritonavir plus two NRTIs [16, 18, 30]. Guidelines recommended viral load (VL) testing 6 months after ART initiation and annually if <1000 copies/ml. For VL ≥1000 copies/ml, repeat VL testing was recommended after 3 months of enhanced adherence counselling. PLWH with second‐line failure (VL ≥1000 copies/ml after 3 months) were recommended assessments of treatment barriers and adherence support. Genotyping was recommended after confirmed second‐line failure despite optimal adherence; genotyping requests were reviewed by a technical working group (TWG) established by the Ministry of Health [16, 18, 30], who provided individualized recommendations based on genotype results; for example continuing the same PI‐based second‐line if no PI resistance, or switching to third‐line or dolutegravir‐based ART (regardless of PI resistance). During the study, Kenya guidelines evolved in “possible” third‐line regimen options (Table S1): 2011, darunavir/ritonavir, raltegravir, etravirine and recycled lamivudine or tenofovir [30]; 2016, combinations of raltegravir (or dolutegravir if available), darunavir/ritonavir, etravirine and lamivudine, with or without another active NRTI [16]; 2018, etravirine removed, and dolutegravir replaced raltegravir, with lamivudine (± another active NRTI) and ± darunavir/ritonavir [18, 31].

AMPATH PLWH >3 years old were eligible for the study if they failed PI‐based second‐line per guidelines (defined above) and underwent genotyping upon second‐line failure. Ethics approval was obtained from Moi University/MTRH (#000391) and Indiana University (#2011632356); informed consent was waived due to the use of routinely collected, de‐identified data.

### 2.2 Data management

We reviewed the medical records of all Resistance Clinic clients to identify all eligible participants. VL testing was performed at the ISO‐accredited AMPATH laboratory. Sanger sequencing of plasma HIV‐1 RNA was performed by the Kisumu Kenya Medical Research Institute and Nairobi National HIV Reference Laboratories [32, 33]. Protease and reverse transcriptase resistance mutations were obtained from sequences requested from laboratories, and when unavailable, from genotyping reports from charts. All participants had no prior integrase strand transfer inhibitor (INSTI) exposure (including raltegravir) and integrase genotyping was not performed. Sequences passing quality control were analysed [34], with subtypes captured from available sequences using REGA [35].

### 2.3 Statistical methods

We summarized clinical, resistance and subtype (when available) data by second‐line PI (atazanavir/ritonavir, lopinavir/ritonavir) at genotyping. Analyses included prevalence of any and class‐specific resistance, and individual mutations. Resistance was interpreted using Stanford tools [36], with antiretroviral (ARV) activity defined by summing penalty scores for each mutation: 0–9 susceptible; 10–14 potential low‐level; 15–29 low‐level; 30–59 intermediate; and ≥60 high‐level resistance. “Any resistance” was defined as any mutation with a score ≥10 (including major and accessory PI mutations). Predicted resistance was estimated to second‐ and third‐line NRTIs, NNRTIs and PIs. We also report rilpivirine resistance, relevant to long‐acting cabotegravir/rilpivirine rollout, though it was not in Kenya's guidelines during the study.

The primary outcome was VF, defined as VL ≥1000 copies/ml, using the first VL 6–18 months post‐genotype among those in care at 6 months post‐genotype, allowing regimen switches based on genotypeto occur (typically within 1–3 months). Subgroups were compared using Fisher exact and Wilcoxon rank sum tests (α = 0.05).

For Hypothesis 1 (H1: post‐genotype VF is similar between switching to third‐line versus remaining on second‐line per guidelines), we compared VF between those on second versus third‐line at 6 months post‐genotype aligned with Kenya guidelines (i.e. “guidelines‐supported regimen”). The TWG interpreted resistance and recommended management on case‐by‐case bases; we defined those with low‐level or higher PI resistance at second‐line failure as on guidelines‐supported regimens if they switched to third‐line or another susceptible second‐line PI. Those without low‐ or higher‐level resistance to their second‐line PI were also considered guideline‐supported if they stayed on their regimen or switched to third‐line (e.g., treatment optimization). Regimens were guidelines‐unsupported if participants remained on their second‐line PI or switched to an alternative second‐line PI, despite low‐ or higher‐level resistance to either PI. Those with ≥2 class resistance who switched to third‐line despite predicted resistance to their third‐line PI (darunavir/ritonavir) or NNRTI (etravirine) drug were also considered guidelines‐supported if regimens contained dolutegravir or raltegravir (assumed susceptibility given no prior INSTI exposure). See File S1 for analytic methods for this and subsequent hypotheses.

For Hypothesis 2 (H2: post‐genotype VF is lower following a guidelines‐supported versus unsupported strategy), we used a Firth model and adjusted for pertinent covariates (gender, years on second‐line, PI at genotyping), since few were on unsupported regimens [37].

For Hypothesis 3 (H3: greater predicted resistance to post‐genotype regimens worsens outcomes regardless of treatment strategy), we used discrete genotypic susceptibility scores (dGSS) to define the extent of predicted resistance to non‐INSTI post‐genotype regimen components and associations with post‐genotype VF, regardless of treatment strategy [38]. We assigned a discrete value of 1 for Stanford scores <30 (susceptible) and 0 for ≥30 (resistant), and summed values for each drug across non‐INSTI components of post‐genotype regimens to create the dGSS. For example, a dGSS = 2 for a tenofovir/lamivudine/dolutegravir regimen, showing high resistance to lamivudine (value = 0), and susceptibility to tenofovir and dolutegravir (value = 1 each).

For Hypothesis 4 (H4: predicted darunavir/ritonavir resistance is similar after atazanavir/ritonavir‐ versus lopinavir/ritonavir‐based second‐line failure), darunavir/ritonavir resistance was defined as penalty score ≥15, and two sub‐analyses were conducted: one defining resistance at Stanford score ≥30 rather than 15; and another using score ≥15 but separating atazanavir/ritonavir users into those with or without prior lopinavir/ritonavir exposure, versus those on lopinavir/ritonavir, accounting for prior lopinavir/ritonavir exposure among those on atazanavir/ritonavir (none on lopinavir/ritonavir had prior atazanavir/ritonavir exposure).

Hypotheses 1, 3 and 4 were examined using odds ratios (OR) with unadjusted logistic regression, inverse probability weighting (IPW) and G‐computation. Our primary analyses used IPW. We report results from G‐computation to assess the sensitivity of the findings to choice of confounder adjustment method. Adjustment variables included calendar year, age, class‐specific resistance, gender, years on PI‐based ART and PI at genotyping.

## 3 RESULTS

**Figure 1:**
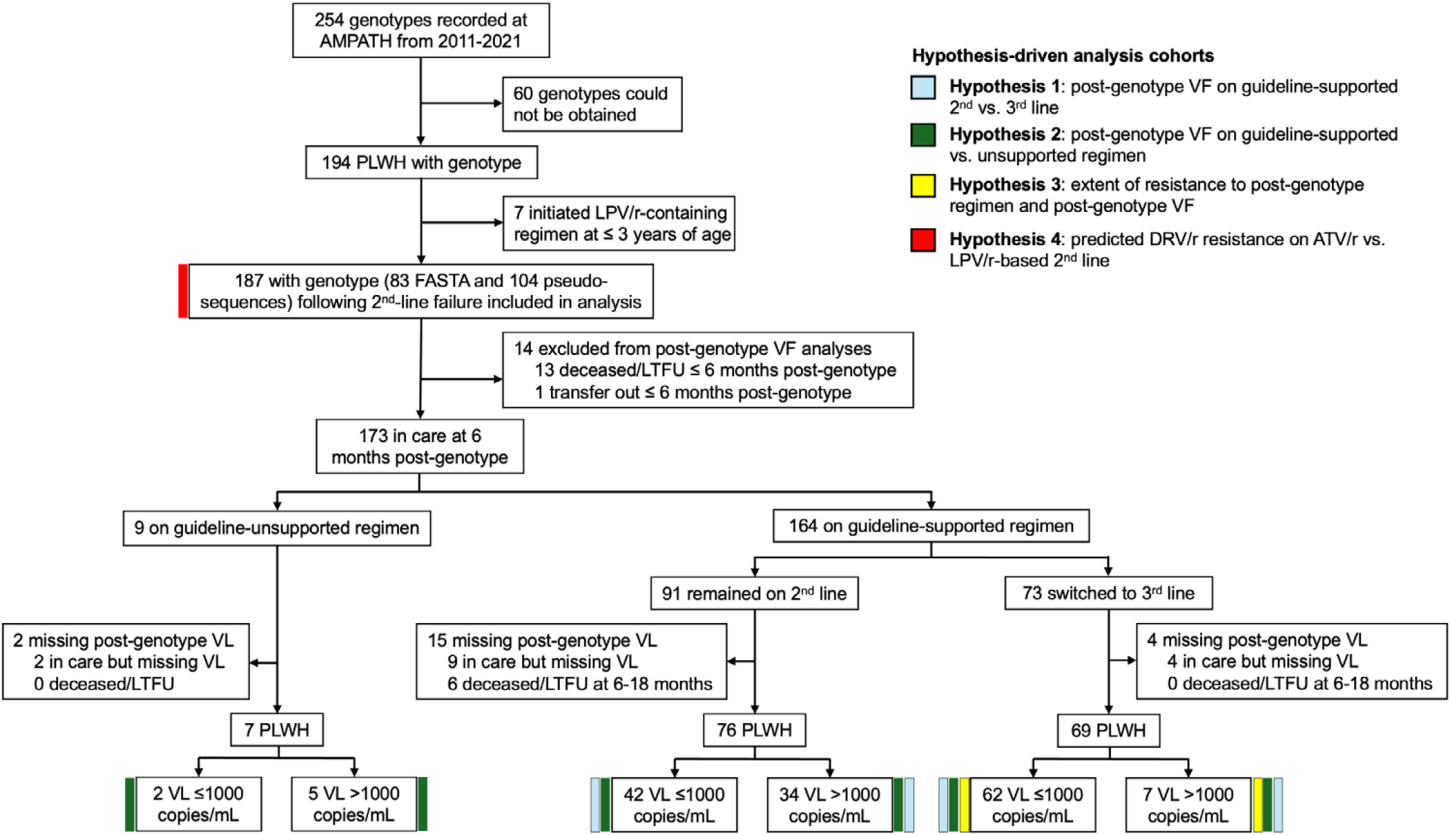
Eligibility flow diagram. Legend: The diagram illustrates the results of the selection process to identify patients with genotyping upon second‐line failure who were included in the overall analysis (*n* = 187), and their viral outcomes. The participants included in each of the hypotheses‐driven analyses are indicated by the coloured lines adjacent to each box, according to the legend (see text for more details). Abbreviations: AMPATH, Academic Model Providing Access to Healthcare; ATV/r, atazanavir/ritonavir; DRV/r, darunavir/ritonavir; LPV/r, lopinavir/ritonavir; LTFU, lost to follow‐up; PLWH, people living with HIV; VF, viral failure; VL, viral load.

A total of 254 second‐line failure genotypes were ordered at AMPATH from 2011 to 2021 (Figure 1). Of these, 60 were unretrievable or unverified and seven were done for children ≤3 years old. Thus, 187 PLWH with genotypes were analysed, with a median age at genotyping 41 years and 54% female (Table 1). The median genotyping year was 2017, earliest in 2011 (before Resistance Clinic started in 2015) and latest in 2021. The median ART duration at genotyping was 9 years, including 4 years on first‐ and 4 years on second‐line. At genotyping, 76/187 were on atazanavir/ritonavir (42% had prior lopinavir/ritonavir exposure) and 111 on lopinavir/ritonavir (none with atazanavir/ritonavir exposure). More were on lopinavir/ritonavir than atazanavir/ritonavir before 2016, when Kenya guidelines first recommended atazanavir/ritonavir over lopinavir/ritonavir for second‐line (Figure S1) [16]. Genotyping reports were available for all participants and sequences for only 83; 62/83 (75%) of whom were subtype A1.

**Table 1:** Characteristics of participants at genotyping performed upon second‐line failure

Variable	Total *N* = 187 *n* (%)	ATV/r *N* = 76 *n* (%)	LPV/r *N* = 111 *n* (%)
Age, median years (range)	41 (6, 70)	43 (16, 70)	40 (6, 68)
Female	101 (54)	40 (53)	61 (55)
Year of genotype, median (range)	2017 (2011, 2021)	2018 (2015, 2021)	2015 (2011, 2021)
Nadir CD4, median cells/mm^3^ (range)	70 (0, 1396)	101 (1, 687)	59 (0, 1396)
Years since nadir CD4, median cells/mm^3^ (range)	7 (0, 17)	8.8 (0, 17)	5 (0, 13)
Years on ART, median (range)	9 (2, 17)	10 (2, 17)	9 (2, 13)
Years on first‐line, median (range)^a^	4 (0.1, 16)	5 (0.1, 12)	3 (0.2, 16)
Years on second‐line, median (range)	4 (0.4, 11)	4 (1, 11)	4 (0.4, 11)
NNRTI exposure pre‐genotype^a^			
EFV only	30 (16)	16 (22)	14 (13)
NVP only	108 (59)	41 (57)	67 (63)
EFV and NVP	41 (23)	15 (21)	26 (24)
PI exposure pre‐genotype			
ATV/r	76 (41)	76 (100)	0 (0)
LPV/r	143 (77)	32 (42)	111 (100)
ART regimen at genotype			
TDF/3TC/ATV/r	56 (30)	56 (74)	–
AZT/3TC/ATV/r	17 (9)	17 (22)	–
Other ATV/r‐containing regimen^b^	3 (2)	3 (4)	–
TDF/3TC/LPV/r	37 (20)	–	37 (33)
ABC/TDF/3TC/LPV/r	24 (13)	–	24 (22)
AZT/3TC/LPV/r	24 (13)	–	24 (22)
ABC/3TC/LPV/r	14 (7)	–	14 (13)
Other LPV/r‐containing regimen^c^	12 (6)	–	12 (11)
HIV‐1 subtype^d^			
A	62 (33)	28 (37)	34 (31)
C	9 (5)	4 (5)	5 (5)
D	6 (3)	2 (3)	4 (4)
G	1 (1)	0 (0)	1 (1)
Other	5 (3)	0 (0)	5 (5)
Missing	104 (56)	42 (55)	62 (56)

Abbreviations: 3TC, lamivudine; ABC, abacavir; ART, antiretroviral therapy; ATV/r, atazanavir/ritonavir; EFV, efavirenz; LPV/r, lopinavir/ritonavir; NNRTI, non‐nucleoside reverse transcriptase inhibitor; NVP, nevirapine; PI, protease inhibitor; TDF, tenofovir disoproxil fumarate.

^a^ Data available for 72 participants on ATV/r and 107 on LPV/r.

^b^ Includes ABC/3TC/ATV/r (*n* = 2) and ABC/TDF/3TC/ATV/r (*n* = 1).

^c^ Includes AZT/TDF/3TC/LPV/r (*n* = 3), ABC/AZT/3TC/LPV/r (*n* = 3), ABC/DDI/3TC/LPV/r (*n* = 3), ABC/AZT/LPV/r (*n* = 2) and ABC/TDF/LPV/r (*n* = 1).

^d^ Subtype available only for those with original sequences; Other subtype category includes AD (*n* = 1), CRF01_AE (*n* = 3) and DA (*n* = 1).

### 3.1 Drug resistance

Most (171/187, 91%) participants had resistance at second‐line failure: 79% NRTI (most common M184V‐73%), 80% NNRTI (most common K103N‐29%) and 37% major PI (most common M46I‐27%) (Table 2 and Figure S2). Dual‐ (36%) or triple‐ (37%) class resistance was present in 73% of participants. NRTI and NNRTI mutation frequencies were similar among those on atazanavir/ritonavir versus lopinavir/ritonavir. However, more major PI mutations were detected upon atazanavir/ritonavir versus lopinavir/ritonavir VF (54% versus 26%) and certain mutations (I50L/V and I84V) differed.

**Table 2:** Genotype results for participants with second‐line failure, stratified by PI at genotyping

Variable	Total *N* = 187 *n* (%)	ATV/r *N* = 76 *n* (%)	LPV/r *N* = 111 *n* (%)
Any resistance^a^	171 (91)	70 (92)	101 (90)
NRTI resistance	148 (79)	60 (79)	88 (79)
NNRTI resistance	150 (80)	62 (82)	88 (79)
PI resistance (major + accessory)	81 (43)	44 (58)	37 (33)
PI resistance (major only)	70 (37)	41 (54)	29 (26)
Intermediate‐high PI resistance^b^	64 (34)	35 (46)	29 (26)
Resistance category^c^			
No resistance	16 (9)	6 (8)	10 (9)
NRTI only	10 (5)	2 (3)	8 (7)
NNRTI only	22 (12)	10 (13)	12 (11)
PI only	1 (1)	0 (0)	1 (1)
NRTI/NNRTI only	58 (31)	14 (18)	44 (40)
NRTI/PI only	10 (5)	6 (8)	4 (4)
NRTI/NNNRTI/PI	70 (37)	38 (50)	32 (29)
ATV/r resistance level^d^			
None or potential low	119 (64)	35 (46)	84 (76)
Low	7 (4)	7 (9)	0 (0)
Intermediate or high	61 (33)	34 (45)	27 (24)
LPV/r resistance level			
None or potential low	126 (67)	44 (58)	82 (74)
Low	6 (3)	6 (8)	0 (0)
Intermediate or high	55 (29)	26 (34)	29 (26)
DRV/r resistance level			
None or potential low	150 (80)	56 (74)	94 (85)
Low	19 (10)	12 (16)	7 (6)
Intermediate or high	18 (10)	8 (11)	10 (9)
ETR resistance level			
None or potential low	103 (55)	40 (53)	63 (57)
Low	16 (9)	9 (12)	7 (6)
Intermediate or high	68 (36)	27 (36)	41 (37)
RPV resistance level			
None or potential low	65 (35)	26 (34)	39 (35)
Low	39 (21)	16 (21)	23 (21)
Intermediate or high	83 (44)	34 (45)	49 (44)

Abbreviations: 3TC, lamivudine; ABC, abacavir; ART, antiretroviral therapy; ATV/r, atazanavir/ritonavir; DRV, darunavir; ETR, etravirine; LPV/r, lopinavir/ritonavir; NNRTI, non‐nucleoside reverse transcriptase inhibitor; NRTI, nucleoside reverse transcriptase inhibitor; NVP, nevirapine; PI, protease inhibitor; RPV, rilpivirine.

^a^ Any resistance is defined as any mutation within each class which conferred a penalty score >10 based on Stanford scores.

^b^ Defined by the Stanford Database HIVDB version 9.1, which was in place at the time of analyses.

^c^ There were no genotypes with NNRTI/PI only resistance.

^d^ Corresponding penalty scores for resistance levels are: none or potential low level, score <15; low level, score 15–29; intermediate or high level, score ≥30.

**Figure 2:**
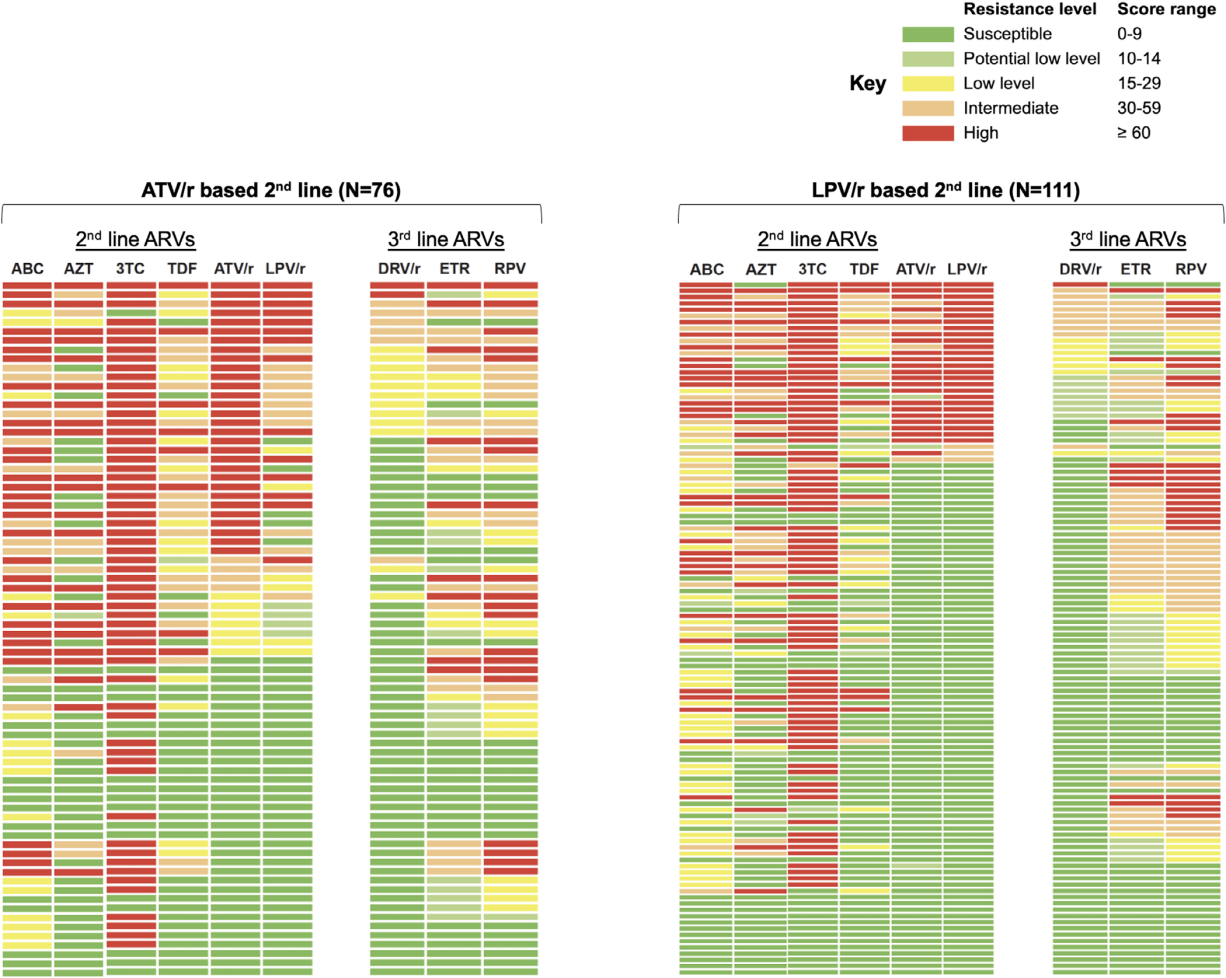
Predicted drug resistance to second‐ and third‐line ARVs, stratified by the second‐line PI at genotyping. Legend: The figure demonstrates two panels, grouped by the second‐line PI at genotyping (ATV/r on the left and LPV/r on the right). Each panel presents detailed information (one row per participant) of one of five predicted levels of drug resistance, according to Stanford Database. The left side of each panel shows predicted resistance to the various second‐line ARVs taken by participants at the time of genotyping, and the right side of each panel shows predicted resistance to potential third‐line ARVs. The rows in each panel are sorted according to the resistance levels (highest to lowest) to the respective second‐line PI at the time of genotyping (i.e. ATV/r resistance among those on ATV/r, and LPV/r resistance among those on LPV/r). The resistance levels and scores are colour‐coded based on the legend at top right of the figure. Abbreviations: 3TC, lamivudine; ABC, abacavir; ARVs, antiretrovirals; ATV/r, atazanavir/ritonavir; AZT, zidovudine; DRV/r, darunavir/ritonavir; ETR, etravirine; LPV/r, lopinavir/ritonavir; RPV, rilpivirine; TDF, tenofovir disoproxil fumarate.

Of 187 participants, 76% had intermediate‐high resistance to ≥1 regimen drug at genotyping (76% NRTIs, 34% PIs, 34% dual‐class) (Figure 2; left side of panels); and 48% had predicted intermediate‐high resistance to ≥1 potential third‐line drug (6% dual‐class) (Figure 2; right side of panels). Overall, 24% had any predicted resistance to darunavir/ritonavir (10% intermediate‐high), 67% to rilpivirine (44% intermediate‐high) and 67% to etravirine (36% intermediate‐high).

Among 76/187 on atazanavir/ritonavir, 46% had intermediate‐high PI resistance (45%‐atazanavir/ritonavir, 34%‐lopinavir/ritonavir, 10%‐darunavir/ritonavir). Among 111/187 on lopinavir/ritonavir, 26% had intermediate‐high PI resistance (26%‐lopinavir/ritonavir, 24%‐atazanavir/ritonavir, 9%‐darunavir/ritonavir). Those on atazanavir/ritonavir with versus without prior lopinavir/ritonavir exposure had similar intermediate‐high atazanavir/ritonavir resistance (50% vs. 44%, *p* = 1.00) and lopinavir/ritonavir resistance (41% vs. 30%, *p* = 0.34).

### 3.2 Guidelines‐supported regimens and post‐genotype VF (H1, H2)

Among 187 participants, 37% had low‐ or higher‐level resistance to their second‐line PI, indicated to switch regimens. Thirteen participants died or were lost to follow‐up (LTFU), and one transferred out within 6 months post‐genotype, resulting in 173 participants in care at 6 months post‐genotype (Figure 1 and Table S2). Among them, 164/173 (95%) were on guidelines‐supported regimens (55% remained on second‐line; 45% switched to third‐line, 86% containing dolutegravir), and of those, 28% had post‐genotype VF (Table S3). Median days from medication switch to VL measurement was 224 (231 for those switched to third‐line, 165 for those switched to alternative second‐line) for those who switched.

Nine (5%) of the 173 were not on guidelines‐supported regimens (all remained on second‐line), and of them, 71% had post‐genotype VF (Figure 1 and Table 3). Thus, 46/152 (30%) participants with post‐genotype VL had VF, after a median of 9 months (range 6–18). None who switched to third‐line died or were LTFU, and 10% had post‐genotype VF. In those staying on guidelines‐supported second‐line, 7% died or were LTFU and 45% had post‐genotype VF. Those switching to third‐line had higher nadir CD4 counts, longer ART duration and lower proportion on lopinavir/ritonavir versus atazanavir/ritonavir at genotyping versus those not switching. In the IPW model for H1 comparing post‐genotype VF on guidelines‐supported second‐ versus third‐line, odds of VF for those switching to third‐line were 93% lower versus those remaining on second‐line (OR = 0.07; 95% CI 0.02−0.20). Logistic regression and G‐computation results were similar (Table 4 and Table S5). In H2 analyses comparing post‐genotype VF on guidelines‐supported versus unsupported regimens, adjusted odds of VF were higher for guidelines‐unsupported versus supported regimens (OR = 4.52; 95% CI 1.02−26.24, Table 4).

**Table 3:** Comparison of participants on guidelines‐supported and unsupported regimens after genotyping, stratified by post‐genotype treatment line among those on guidelines‐supported regimens

	Guidelines‐supported regimen			Guidelines‐unsupported regimen
Variable	Total *N* = 173 *n* (%)	Switched to third‐line *N* = 73 *n* (%)	Stayed on second‐line *N* = 91 *n* (%)	*N* = 9 *n* (%)
Age, median years (range)	41 (6, 70)	41 (6, 70)	41 (6, 68)	44 (14, 66)
Female	94 (54)	35 (48)	57 (63)	2 (22)
Nadir CD4, median cells/mm^3^ (range)	79 (0, 1396)	114 (0, 1396)	62 (1, 935)	34 (3, 255)
Years since nadir CD4, median cells/mm^3^ (range)	7 (0, 17)	8 (0, 17)	6 (0, 14)	6 (1, 12)
Years on ART, median (range)	9 (2, 17)	10 (2, 17)	8 (2, 13)	12 (5, 14)
Years on second‐line, median (range)	4 (0.4, 11.4)	4 (1, 11)	4 (0.4, 11)	5 (1, 9)
Year of genotype, median (range)	2017 (2011, 2021)	2019 (2015, 2021)	2015 (2011, 2021)	2015 (2011, 2020)
ART regimen at genotype				
TDF/3TC/ATV/r	51 (29)	35 (48)	15 (16)	1 (11)
TDF/3TC/LPV/r	35 (20)	10 (14)	23 (25)	2 (22)
TDF/3TC/ABC/LPV/r	23 (13)	1 (1)	20 (22)	2 (22)
AZT/3TC/LPV/r	22 (13)	8 (11)	14 (15)	0 (0)
AZT/3TC/ATV/r	15 (9)	12 (16)	3 (3)	0 (0)
ABC/3TC/LPV/r	13 (8)	7 (10)	4 (4)	2 (22)
Other ATV/r‐containing regimen^a^	3 (2)	0 (0)	3 (3)	0 (0)
Other LPV/r‐containing regimen^b^	11 (6)	0 (0)	9 (10)	1 (22)
PI at genotype				
ATV/r	69 (40)	47 (64)	21 (23)	1 (11)
LPV/r	104 (60)	26 (36)	70 (77)	8 (89)
Second‐line regimen at 6 months post‐genotype	–			
TDF/3TC/LPV/r	22 (13)	–	21 (23)	1 (11)
TDF/3TC/ATV/r	23 (13)	–	21 (23)	2 (22)
TDF/3TC/ABC/LPV/r	19 (11)	–	16 (18)	3 (33)
AZT/3TC/LPV/r	11 (6)	–	11 (12)	0 (0)
AZT/3TC/ATV/r	4 (2)	–	4 (4)	0 (0)
Other second‐line regimen^c^	21 (12)	–	18 (20)	3 (33)
Third‐line regimen at 6 months post‐genotype				–
TDF/3TC/DTG	–	30 (41)	–	–
TDF/3TC/DTG/DRV/r	–	20 (27)	–	–
AZT/3TC/DTG	–	4 (5)	–	–
TDF/3TC/DTG/DRV/r	–	3 (4)	–	–
AZT/3TC/DTG/DRV/r	–	2 (3)	–	–
ETR/RAL/DRV/r	–	2 (3)	–	–
TDF/3TC/DRV/r	–	2 (3)	–	–
Other third‐line regimen^d^	–	10 (14)	–	–
Outcome post‐genotype				
VL ≤ 1000 copies/ml	106 (61)	62 (85)	42 (46)	2 (22)
VL > 1000 copies/ml	46 (27)	7 (10)	34 (37)	5 (56)
Deceased or LTFU	6 (3)	0 (0)	6 (7)	0 (0)
Missing^e^	15 (9)	4 (5)	9 (10)	2 (22)

Abbreviations: 3TC, lamivudine; ABC, abacavir; ART, antiretroviral therapy; ATV/r, atazanavir/ritonavir; DRV, darunavir; ETR, etravirine; LPV/r, lopinavir/ritonavir; LTFU, lost to follow‐up; PI, protease inhibitor.

^a^ Includes ABC/3TC/ATV/r (*n* = 2) and TDF/3TC/ABC/ATV/r (*n* = 1).

^b^ Includes TDF/3TC/AZT/LPV/r (*n* = 3), AZT/3TC/ABC/LPV/r (*n* = 2), ABC/DDI/3TC/LPV/r (*n* = 3) and ABC/AZT/LPV/r (*n* = 2).

^c^ For those on a guidelines‐supported regimen who stayed on second‐line, “other” includes TDF/3TC/AZT/LPV/r (*n* = 3), ABC/3TC/LPV/r (*n* = 5), ABC/3TC/DDI/LPV/r (*n* = 1), ABC/AZT/3TC/LPV/r (*n* = 3), TDF/3TC/ATV/r (*n* = 3), ABC/AZT/LPV/r (*n* = 2), D4T/3TC/LPV/r (*n* = 1), TDF/3TC/ATV/r (*n* = 1), ABC/3TC/ATV/r (*n* = 1) and TDF/3TC/ATV/r (*n* = 1). For those on a guidelines‐unsupported regimen, “other” includes ABC/3TC/LPV/r (*n* = 2) and ABC/DDI/3TC/LPV/r (*n* = 1).

^d^ Includes *n* = 1 each of 3TC/TDF/RAL/DRV/r, DTG/3TC/DRV/r, TDF/3TC/ETR/RAL/DRV/r, TDF/3TC/RAL, AZT/3TC/RAL/DRV/r, TDF/3TC/DTG/ATV/r, AZT/3TC/RAL, 3TC//ETR/DTG/DRV/r, TDF/3TC/ETR/DRV/r and ABC/3TC/DTG/DRV/r.

^e^ Missing include those in care through 18 months but missing a VL within 6–18 months.

**Table 4:** Associations between drug resistance, guidelines strategy and VF according to hypotheses 1–4

	Odds ratios and 95% confidence intervals	
Analytic approach	Inverse probability weighting	Unadjusted logistic regression
Hypothesis 1 (VF by third‐line switch vs. staying on second‐line)	0.07 (0.02, 0.20)	0.14 (0.04, 0.32)
Hypothesis 2 (VF by inappropriate switch vs. guidelines switch)^a^	–	5.54 (1.28, 31.82)
Hypothesis 3 (VF for one higher genotype susceptibility)	0.82 (0.52, 1.35)	0.89 (0.57, 1.48)
Hypothesis 4 (ATV vs. LPV)		
Outcome: low or higher‐level predicted DRV/r resistance		
ATV/r versus LPV/r	0.96 (0.35, 2.75)	1.86 (0.89, 4.01)
Outcome: low or higher‐level predicted DRV resistance		
ATV/r only versus LPV/r only	1.47 (0.32, 6.72)	1.66 (0.61, 3.90)
LPV/r → ATV/r versus LPV/r only	1.06 (0.31, 3.43)	2.17 (0.75, 5.45)
Outcome: intermediate‐high level predicted DRV/r resistance		
ATV/r versus LPV/r	0.94 (0.34, 2.56)	1.85 (0.87, 3.98)

Abbreviations: ATV/r, atazanavir/ritonavir; DRV/r, darunavir/ritonavir; LPV/r, lopinavir/ritonavir; VF, viral failure.

^a^ Only nine participants were given a guidelines‐unsupported treatment regimen, these estimates are from a Firth model and IPW was not done.

### 3.3 Resistance and post‐genotype VF (H3, H4)

H3 goal is to examine whether resistance to post‐genotype regimens measured by dGSS is associated with post‐genotype VF. To control for potential confounding by regimen line, we included regimen line (second or third) as an adjustment covariate. Regimen line together with dGSS determines whether the regimen is guideline‐supported; hence, it is unnecessary to further adjust for whether regimens follow guidelines. Among those switching to third‐line (*n* = 73), 1% had dGSS = 0, 19% dGSS = 1, 51% dGSS = 2, 27% dGSS = 3 and 1% dGSS = 4 (combined with dGSS = 3 for this analysis) (Table S4). In the IPW analysis, VF odds were similar regardless of predicted resistance (OR = 0.82 for one higher predicted level of resistance; 95% CI 0.52−1.35) (Table 4). ORs from logistic regression and G‐computation analyses were similar (Table S5).

The H4 analysis comparing predicted darunavir/ritonavir resistance among those on atazanavir/ritonavir versus lopinavir/ritonavir at genotyping included 187 participants with genotypes. Overall, 10% had intermediate‐high darunavir/ritonavir predicted resistance (9% on atazanavir/ritonavir, 10% on lopinavir/ritonavir). In the IPW analysis, darunavir/ritonavir resistance was similar among the two groups (OR = 0.96, 95% CI 0.35−2.75 for resistance score ≥15; OR = 0.94, 95% CI 0.34−2.56 for score ≥30) (Table 4). ORs from logistic regression and G‐computation analyses were similar (Table S5). Using IPW to compare those on atazanavir/ritonavir with and without past lopinavir/ritonavir exposure to those on lopinavir/ritonavir also showed no significant association with darunavir/ritonavir resistance.

## 4 DISCUSSION

We report extensive drug resistance and concerning early viral outcomes among ART‐experienced Kenyan PLWH following PI‐based second‐line failure. Guidelines compliance was good but imperfect and viral outcomes were better when guidelines were followed, particularly when switching to third‐line after second‐line failure. Findings highlight the vulnerability of PLWH with second‐line failure, 24% of whom were <25 years old requiring lifelong ART with close monitoring. As dolutegravir access expands globally, PIs remain important alternatives, and programmatic data are critical to identify resistance patterns in new regimens and treatment sequences, guiding care strategies.

In our study, >90% of PLWH had drug resistance upon second‐line failure, exceeding the 70–80% reported in other African settings [2, 3, 4, 7, 39, 40]. Triple‐class resistance was 37%, exceeding the 7–25% seen elsewhere in Africa [3, 7, 41, 42, 43], but comparable to South Africa and Namibia (30−40%) [4, 8]. Differences in populations, subtypes, adherence or tolerability, boosted/unboosted PIs, monitoring and treatment durations may explain this heterogeneity [2, 8]. Our heavily treatment‐experienced cohort, potentially biased due to referrals to the Drug Resistance Clinic, might also contribute to high observed resistance. Genotyping may be limited to those at highest risk, entailing under‐detection of resistance in the population. Importantly, NRTI cross‐resistance can affect newer drugs, regimens and formulations even without major or any PI resistance, underscoring the need for early resistance testing upon second‐line failure and close follow‐up, including after transitioning to dolutegravir‐based ART, for this vulnerable population.

Predicted resistance to key third‐line agents at second‐line failure is concerning and underscores the importance of resistance testing in regimen design. Ten percent of participants had intermediate‐high darunavir/ritonavir resistance, versus 3–8% in other LMICs, supporting darunavir's role as a third‐line anchor drug, with/without dolutegravir [3, 4, 39, 44]. Predicted intermediate‐high resistance to etravirine (36%) and rilpivirine (44%), likely related to prior NNRTI exposure and potentially underestimated due to mutation decay over time [45], is comparable to other African cohorts (22−45% and 23–43%, respectively) [3, 7, 39, 44]. As the long‐acting cabotegravir/rilpivirine rollout progresses, the high risk of rilpivirine resistance, even following second‐line failure, is a critical concern. These results are likely influenced by limited VL and drug resistance monitoring typical in many LMICs, leading to resistance accumulation and cross‐resistance. This situation demands thorough follow‐up, including understanding archived and circulating resistance across all classes upon PI‐based second‐line failure, especially important when considering dolutegravir and darunavir/ritonavir for third‐line and beyond in individuals with advanced treatment histories and complex resistance profiles.

Predicted darunavir/ritonavir resistance was similar upon failure of atazanavir/ritonavir and lopinavir/ritonavir‐based regimens despite higher PI resistance upon atazanavir/ritonavir (54%) versus lopinavir/ritonavir (26%) failure, and some prior lopinavir/ritonavir exposure in the former group. ACTG A5228 also reported higher PI resistance upon atazanavir/ritonavir (46%) versus lopinavir/ritonavir (30%) failure [39]. Higher pill burdens and side effects of lopinavir/ritonavir versus atazanavir/ritonavir possibly worsened lopinavir/ritonavir adherence, resulting in less resistance [46, 47]. These results offer some reassurance as to the equivalence of lopinavir/ritonavir and atazanavir/ritonavir exposure regarding predicted darunavir/ritonavir resistance and their sequential use in some settings.

Post‐genotype VF after a median of 9 months occurred in 30% of participants with VL data regardless of treatment strategy. This is alarmingly high but within the 5–36% rates in other LMICs [2, 3, 48]. Post‐genotype VF was substantially lower among those on guidelines‐supported (41/145, 28%) versus unsupported regimens (5/7, 71%), highlighting the importance of complying with guidelines. The 95% CI (1.2−31.8) indicates considerable uncertainty about the true magnitude of this effect, and this result should be interpreted with appropriate caution. Still, although only 5% of participants were on guidelines‐unsupported regimens, their poorer outcomes invoke the need to assess and monitor guidelines compliance, as done for HIV testing and ART initiation [49, 50, 51, 52]. This is imperative for individuals with advanced ART histories and resistance profiles, especially after second‐ or third‐line VF and beyond. Monitoring guidelines compliance is particularly relevant in the new era of dolutegravir‐based ART to inform strategies to enhance care.

We found 87% lower odds of post‐genotype VF after switching to third‐line versus staying on second‐line, within guidelines‐recommended strategies. In ACTG A5288, post‐genotype VF was 66% in adults without lopinavir/ritonavir resistance maintained on lopinavir/ritonavir‐based second‐line, versus ≤26% in those switched to third‐line [48]. A Zimbabwe study reported similar results [2]. Factors driving these outcomes were not examined here but may include poor second‐line adherence, superior third‐line (mostly dolutegravir‐based) efficacy, tolerability and resistance barriers, or clinic‐level factors like intensified services in a dedicated clinic for clients on their “last ART line” [53, 54, 55]. Close monitoring and support are essential for individuals remaining on PI‐based second‐line after failure, even if guidelines‐compliant without PI resistance, with consideration of transitioning to third‐line. Indeed, PI resistance could develop after genotyping and before subsequent VL measurement. Although PIs including darunavir/ritonavir have high resistance barriers, rapid accumulation of protease mutations can occur following initial PI resistance [7, 8].

Despite better outcomes after switching to third‐line, 63% of participants remained susceptible to their second‐line PI at failure. This is consistent with other studies, likely reflecting poor second‐line adherence or intolerability, and supports switching to dolutegravir‐based ART even without PI resistance [3, 8, 39]. Given the challenges distinguishing poor and non‐adherence clinically, even with emerging drug‐level technologies, genotyping remains crucial for guiding management [56]. We found no associations between predicted resistance to the post‐second‐line failure regimen and subsequent VF, possibly due to small sample size, or the improved efficacy and tolerability of third‐line regimens, clinical support and understanding of advanced treatment stages. This should not undermine the consideration of resistance testing, particularly during advanced treatment stages where it remains essential for regimen design. This includes consideration of alternative resistance mechanisms and mutations outside the protease region (e.g., *gag* or *env*) undetected by routine assays and minority drug resistance variants [57, 58]. A better understanding of second‐line failure and the utility of switching to dolutegravir‐ or darunavir/ritonavir‐based ART, with or without PI and other resistance, is needed.

Study strengths include the focus on real‐world outcomes, compliance with guidelines and comprehensive analyses. Limitations include missing adherence data and related interventions, and the time since initial viraemia detection to confirmed VF and subsequent clinical actions which may have influenced management and outcomes. Despite efforts to retrieve all genotypes, we likely underrepresented AMPATH second‐line failures, highlighting the need for routine genotype curation for care and research.

## 5 CONCLUSIONS

We found high drug resistance levels and early VF after second‐line PI‐based failure in Kenya. Outcomes were better with guidelines compliance and after switching to third‐line, even within guidelines recommendations and without PI resistance. Findings, cautiously interpreted, underscore the importance of complying with guidelines, ongoing research to reevaluate them and the need for better third‐line access, drug resistance testing, and understanding resistance mechanisms in highly treatment‐experienced individuals in the new ART era. Better adherence monitoring and support are also needed to identify and address psychosocial and structural adherence barriers. Given the continued role of PIs in the new ART era as alternatives or complements to dolutegravir in treatment guidelines, understanding resistance patterns upon failure of PI‐based ART remains important. Continued programmatic drug resistance surveillance is crucial to ensure guidelines compliance, enhance monitoring and support, and determine optimal clinical strategies for managing second‐line failure.

## COMPETING INTERESTS

The authors report no competing interests.

## AUTHORS’ CONTRIBUTIONS

JMH, SMA, AD, JWH and RK conceptualized and designed the study and had primary responsibility for the interpretation of the data. JMH, SMA and RK were responsible for regulatory approval. JMH, SMA, ES, BJ, EK, CN and SG collected the data. JMH, AD, VN and JWH analysed the data. JMH wrote the paper with assistance from all coauthors. All authors have read and approved the final manuscript.

## FUNDING

This project was funded with support from the National Institutes of Health grants K24AI134359 and P30AI042853.

## DISCLAIMER

The content is solely the responsibility of the authors and does not necessarily represent the official views of the National Institutes of Health.

## Supporting information




**Supplement File 1**: jia226523‐sup‐0001‐SuppMat.docx

**Figure S1** *Title*: Distribution of genotyping performed upon 2^nd^‐line failure over time, by 2^nd^‐line PI exposure at genotyping
**Figure S2**. *Title*: Frequency of NRTI, NNRTI and PI HIV‐1 drug resistance mutations in 187 participants with genotypes following failure of 2^nd^‐line ART
**Table S1**. Summary of evolving Kenya Guidelines for possible 3^rd^‐line ART in children, adolescents and adults during the study period, 2011‐2021
**Table S2**. Characteristics of participants by (A) whether guidelines recommended switch from 2^nd^‐line based on genotype results, and (B) retention status at 6 months post‐genotype.
**Table S3**. Characteristics of participants with a guidelines‐supported treatment strategy after genotyping, by post‐genotype treatment line and viral load availability, respectively.
**Table S4**. Predicted resistance to the post‐genotype regimen stratified by whether the post‐genotype treatment strategy was in accordance with the guidelines.
**Table S5**. Sensitivity analysis evaluating the associations between drug resistance, guidelines strategy, and VF according to hypotheses 1, 3 and 4^a^. Odds Ratios and 95% Confidence Intervals from G‐computation.

## Data Availability

The data that support the findings of this study are available from the corresponding author upon reasonable request.
