# Supplementary material for: HIV drug resistance, early treatment outcomes and impact of guidelines compliance after protease inhibitor‐based second‐line failure in a dedicated resistance clinic in western Kenya: a retrospective cohort study
Source: J Int AIDS Soc. 2025 Jun 9;28(6):e26523. doi: 10.1002/jia2.26523 (PMC12148949; doi:10.1002/jia2.26523)
Supplement: Supplementary file 1 — Supplement File 1: jia226523‐sup‐0001‐SuppMat.docx [file JIA2-28-e26523-s002.docx]

**SUPPORTING INFORMATION FILE 1**

### **Supplemental Analysis Text**

While the actual exposure and binary outcome vary by hypothesis, we use the same general statistical approach to evaluate hypotheses 1, 3 and 4. For each hypothesis, we begin by estimating the effects of the exposure by first fitting a model of the form

$logit\left\{ P\left( Y=1 | X, A \right) \right\}=\mu+A\theta+X\beta$, (1)

where Y denotes the binary outcome status (1=yes, 0=no), X represents the vector of subject-specific adjustment variables and A is the exposure. For hypotheses 1 and 3 the binomial outcome is treatment failure at 6 months, and for hypothesis 4, the outcome is resistance to darunavir. The exposure (A) for hypothesis 1 is a binomial indicator variable for switching to 3^rd^-line after genotyping; the exposures (A) for hypothesis 3 are an ordinal measure of predicted resistance or genotype susceptibility to the treatment assigned after genotyping; and the exposure (A) for hypothesis 4 is either a binomial indicator variable for being on ATV (yes/no) at the time of the genotype, or a 3-level factor variable whereby those on ATV are further stratified by whether they had ever been exposed to LPV.

The model parameters, μ, θ and β, are estimated using both unweighted and weighted logistic regression.

We estimate stabilized inverse probability weights for each observation, *i*, in the dataset using the ipw R package (van der Wal et al.). The weights are estimated similarly for binomial, ordinal or polytomous exposures, A. The stabilized weights are calculated as:

${sw}_{i}=\frac{P\left( A_{i}=a_{i} \right)}{P\left( A_{i}=a_{i} | X_{i}=x_{i} \right)},$ (2)

where the numerator is the estimated probability participant *i* receives their observed exposure level, *a_i_*, using the appropriate modeling approach based on the distribution of the exposure. Discrete exposures are modeled using logistic regression, ordinal exposures using proportional odds ordinal logistic regression, and polytomous exposures using multinomial regression. The denominator is the probability participant *i* receives their observed exposure level, *a_i,_*, conditional on the observed values of participant *i*’s covariates, *X_i_*. The observations with weights outside the observed 1^st^ and 99^th^ percentile are truncated to the value of the observed 1^st^ and 99^th^ percentiles.

When fitting the outcome and weight models, we allow the continuous covariates, X, to be modeled with a flexible, nonlinear spline and/or a categorized version of the continuous measure. The best functional form is determined as the one resulting in the lowest AIC when fit to the full observed data.

Due to the large number of potentially important covariates, *X*, as listed in the main paper, and to avoid overfitting, we use backwards model selection prior to capturing θ and estimating the weights. Bootstrap resampling was used to estimate the confidence intervals about the mean risk difference (RD) or odds ratio (OR). We created 5000 sampled datasets from the study data, sampling individuals with replacement. For each bootstrap sample, we first fit the weight model (2) and then the outcome model (1) so that the bootstrap accounts for uncertainty in both models.

Finally, for each exposure and outcome we provide treatment effects 3 different ways, specifically:

1. Unadjusted/unweighted: estimate Model (1) unweighted and unadjusted (assume β=0).
2. Unadjusted/weighted: like (a) but weighted using stabilized weights from Model (2).
3. G-computation: use Model (1) weighted using weights from Model (2) and expand the covariates to include interactions between the exposure (A) and a covariate (X) when the covariate is present in both Model (1) and model (2) after model selection. To implement the G-computation algorithm, we generated the predicted probability of the outcome for each participant under each of the possible exposure groups by plugging in the participant’s covariates into model (1) and setting the treatment group variables, A, to fixed values corresponding to the distinct levels (e.g. as described by Moore et al). Next, we calculated the covariate-adjusted estimator $\hat{P}\left( Y=1 | A=a \right)$ as the sample mean of predicted probabilities corresponding to treatment A=a. The probability is calculated by averaging the predicted values; i.e.,

$\hat{P}\left( Y=1 | A=a \right)=\frac{1}{n}\sum_{i=1}^{n} \hat{P}\left( Y=1 | A=a, x_{i} \right)$ (3)

Treatment group comparisons were made using the risk difference compared to the reference exposure level, represented by *a*=0.

The estimated risk difference is calculated as:

$\hat{RD}=\hat{P}\left( Y=1 | A=a \right)-\hat{P}\left( Y=1 | A=0 \right)$,

where *a* is set to correspond to each of the possible exposure levels. Similarly, the odds ratio is calculated via:

$\hat{OR}=\frac{{\hat{P}\left( Y=1 | A=a \right)}/\left( 1-\hat{P}\left( Y=1 | A=a \right) \right)}{{\hat{P}\left( Y=1 | A=0 \right)}/\left( 1-\hat{P}\left( Y=1 | A=0 \right) \right)}$,

Under the assumption of no unmeasured confounders, the estimate in (3) corresponds to the probability of the outcome under the scenario that all individuals received exposure A=a. Hence contrasts, such as risk differences and odds ratios, can be interpreted as causal effects.

**References**

van der Wal and Geskus. 2011. ipw: An R Package for Inverse Probability

Weighting. Journal of Statistical Software. September 2011, Volume 43, Issue 13. http://www.jstatsoft.org/

Moore KL, Neugebauer R, Valappil T, Laan MJ. Robust extraction of covariate information to improve estimation efficiency in randomized trials. Stat Med. 2011 Aug 30;30(19):2389-408. doi: 10.1002/sim.4301. Epub 2011 Jul 12. PMID: 21751231; PMCID: PMC4113477.
