# Supplementary material for: HIV drug resistance, early treatment outcomes and impact of guidelines compliance after protease inhibitor‐based second‐line failure in a dedicated resistance clinic in western Kenya: a retrospective cohort study
Source: J Int AIDS Soc. 2025 Jun 9;28(6):e26523. doi: 10.1002/jia2.26523 (PMC12148949; doi:10.1002/jia2.26523)
Supplement: Supplementary file 2 — Figure S1 Title: Distribution of genotyping performed upon 2nd‐line failure over time, by 2nd‐line PI exposure at genotyping Figure S2. Title: Frequency of NRTI, NNRTI and PI HIV‐1 drug resistance mutations in 187 participants with genotypes following failure of 2nd‐line ART Table S1. Summary of evolving Kenya Guidelines for possible 3rd‐line ART in children, adolescents and adults during the study period, 2011‐2021 Table S2. Characteristics of participants by (A) whether guidelines recommended switch from 2nd‐line based on genotype results, and (B) retention status at 6 months post‐genotype. Table S3. Characteristics of participants with a guidelines‐supported treatment strategy after genotyping, by post‐genotype treatment line and viral load availability, respectively. Table S4. Predicted resistance to the post‐genotype regimen stratified by whether the post‐genotype treatment strategy was in accordance with the guidelines. Table S5. Sensitivity analysis evaluating the associations between drug resistance, guidelines strategy, and VF according to hypotheses 1, 3 and 4a. Odds Ratios and 95% Confidence Intervals from G‐computation. [file JIA2-28-e26523-s001.docx]

**SUPPORTING INFORMATION FILE 2**

**Figure S1. *Title*: Distribution of genotyping performed upon 2^nd^-line failure over time, by 2^nd^-line PI exposure at genotyping.**

*Legend:* The figure details the frequency percentages (Y axis) of the 2^nd^-line PI each of the 187 participants were on at time of genotyping, stratified by the year in which genotyping was performed (X axis). Grey bars represent those on ATV/r without prior LPV/r exposure; green bars represent those on LPV/r without prior ATV/r exposure; orange bars represent those on ATV/r with prior LPV/r exposure. Abbreviations: ATV/r, atazanavir/ritonavir; LPV/r, lopinavir/ritonavir.


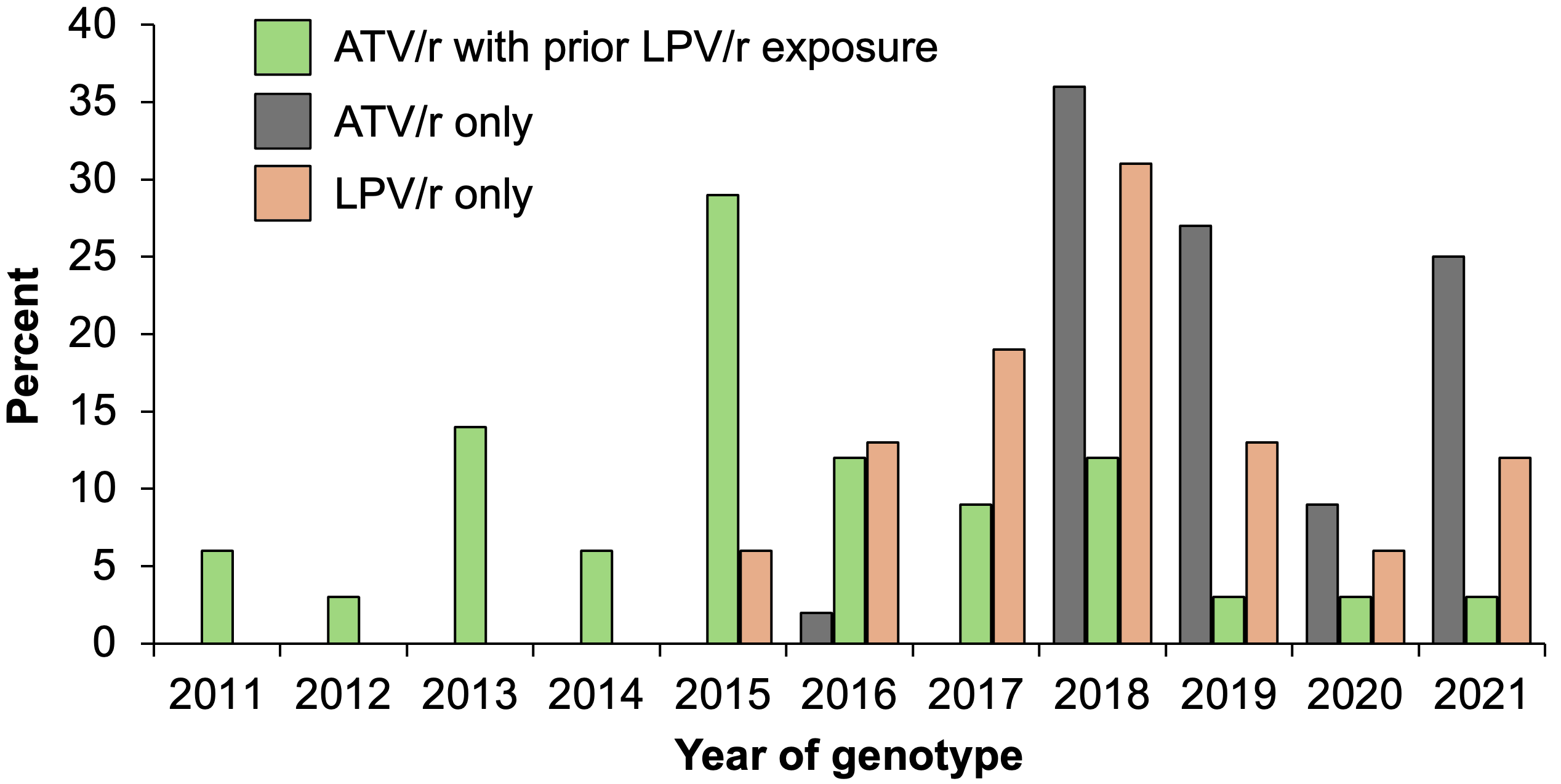


**Figure S2. *Title:* Frequency of NRTI, NNRTI and PI HIV-1 drug resistance mutations in 187 participants with genotypes following failure of 2^nd^-line ART.**

*Legend:* Each figure details the frequency percentages (Y axis) of mutations associated with resistance (X axis) to NRTIs (panel A), NNRTIs (panel B) and PIs (panel C) for 187 PLWH, sorted by mutation frequency in descending order and stratified by the 2^nd^-line PI at genotyping (see legend). Abbreviations: ATV, atazanavir; LPV, lopinavir; NRTI nucleoside reverse transcriptase inhibitor; NNRTI, non-nucleoside reverse transcriptase inhibitor; PI protease inhibitor.

1. NRTI mutation frequency

1. NNRTI mutation frequency

1. PI major mutation frequency

**Table S1. Summary of evolving Kenya Guidelines for possible 3^rd^-line ART in children, adolescents and adults during the study period, 2011-2021.**

| **2011 Guidelines*** **[**[**30**](#_ENREF_30)**]** | **2016 Guidelines [**[**16**](#_ENREF_16)**]** | **2018 Guidelines [**[**18**](#_ENREF_18)**]** |
| --- | --- | --- |
| **Children** | | |
| No specific guidance | raltegravir (or dolutegravir) + lamivudine + darunavir/ritonavir | same |
|  | zidovudine + raltegravir (or dolutegravir) + lamivudine + darunavir/ritonavir | same |
|  | abacavir/tenofovir + raltegravir (or dolutegravir) + lamivudine + darunavir/ritonavir | same |
|  | etravirine + lamivudine + darunavir/ritonavir | same |
| **Adolescents and Adults** | | |
| **Recommended 3^rd^-line drugs:**   - darunavir/ritonavir - raltegravir - etravirine - Recycling drugs that confer benefit: lamivudine, tenofovir | raltegravir (or dolutegravir) + lamivudine + darunavir/ritonavir | dolutegravir + lamivudine + darunavir/ritonavir |
|  | zidovudine + raltegravir (or dolutegravir) + lamivudine + darunavir/ritonavir | dolutegravir + zidovudine + lamivudine + darunavir/ritonavir |
|  | tenofovir + raltegravir (or dolutegravir) + lamivudine + darunavir/ritonavir | dolutegravir + tenofovir + lamivudine + darunavir/ritonavir |
|  | etravirine + lamivudine + darunavir/ritonavir | same |
|  | -- | dolutegravir + tenofovir (or zidovudine) + lamivudine |

Footnote: * Comments in the Guideline: (1) Third-line regimens should contain at least two fully active drugs for durable, potent virologic suppression. (2) Third-line regimen choice must be guided by resistance testing. (3) Based on current standardized treatment regimens, majority of patients are likely to achieve full virologic suppression with a regimen of darunavir/ritonavir plus raltegravir plus lamivudine +/- tenofovir. (4) Patients who failed an initial NNRTI based first line regimen are unlikely to benefit from Etravirine, as part of the third line treatment. The use of Etravirine should therefore be guided by further understanding of NNRTI resistance at first line failure. (5) Lamivudine associated resistant viruses have poor replication capacity compared to wild type virus and should be maintained in patients requiring third-line regimens. (6) Patients failing TDF may still benefit from continuation of TDF despite the presence of the characteristic TDF mutations. (7) Based on current standardized treatment regimens, majority of patients are likely to achieve full virologic suppression with a regimen of darunavir/ritonavir plus raltegravir plus lamivudine plus tenofovir. (8) Maintain the patient on the failing regimen until a full third-line regimen is available as recommended by the national TWG.

**Table S2. Characteristics of participants by (A) whether guidelines recommended switch from 2^nd^-line based on genotype results, and (B) retention status at 6 months post-genotype.**

| **Variable** | **A. Guidelines recommended switch from 2^nd-^line** | | **B. Retention status at 6 months post-genotype** | | |
| --- | --- | --- | --- | --- | --- |
|  | **Switch indicated**  **N=70 n (%)** | **Switch not indicated N=117 n (%)** | **In care**  **N=173 n (%)** | **Not in care**  **N=14 n (%)** | **P value** |
| Age, median (range) | 43 (6, 70) | 40 (6, 68) | 41 (6, 70) | 41 (17, 58) | 0.60 |
| Female | 29 (41) | 72 (62) | 94 (54) | 7 (50) | 0.79 |
| Nadir CD4, median (range) | 56 (0, 1280) | 79 (1, 1396) | 79 (0, 1396) | 19 (1, 296) | 0.02 |
| Years since nadir CD4, median (range) | 8 (0, 17) | 7 (0, 14) | 7 (0, 17) | 8 (0, 13) | 0.75 |
| Years on ART, median (range) | 10 (2, 17) | 9 (2, 13) | 9 (2, 17) | 8 (4, 13) | 0.80 |
| Years on 2^nd^-line, median (range) | 4 (0.5, 11) | 4 (0.4, 11) | 4 (0.4, 11) | 4 (1, 7) | 0.39 |
| Year of genotype, median (range) | 2018 (2011, 2021) | 2016 (2011, 2021) | 2017 (2011, 2021) | 2016 (2013, 2020) | 0.21 |
| ART regimen at genotype |  |  |  |  |  |
| TDF/3TC/ATV/r | 31(44) | 25 (21) | 51 (29) | 5 (36) | 0.85 |
| TDF/3TC/LPV/r | 13 (19) | 24 (21) | 35 (20 | 2 (14) |  |
| ABC/TDF/3TC/LPV/r | 2 (3) | 22 (19) | 23 (13) | 1 (7) |  |
| AZT/3TC/LPV/r | 3 (4) | 21 (18) | 22 (13) | 2 (14) |  |
| AZT/3TC/ATV/r | 9 (13) | 8 (7) | 15 (9) | 2 (14) |  |
| ABC/3TC/LPV/r | 8 (11) | 6 (5) | 13 (8) | 1 (7) |  |
| Other ATV/r regimen^a^ | 1 (1) | 2 (2) | 3 (2) | 0 (0) |  |
| Other LPV/r regimen^b^ | 3 (4) | 9 (8) | 11 (6) | 1 (7) |  |
| PI at genotype |  |  |  |  |  |
| ATV/r | 41 (59) | 35 (30) | 69 (40) | 7 (50) | 0.57 |
| LPV/r | 29 (41) | 82 (70) | 104 (60) | 7 (50) |  |
| Any PI resistance | 70 (100) | 11 (9) | 77 (45) | 4 (29) | 0.28 |
| Any NRTI resistance | 70 (100) | 78 (67) | 138 (80) | 10 (71) | 0.50 |
| Any NNRTI resistance | 62 (89) | 88 (75) | 140 (81) | 19 (71) | 0.48 |

Footnote: ^a^ Includes ABC/3TC/ATV (n=2) and ABC/TDF/3TC/ATV (n=1). ^b^ Includes AZT/TDF/3TC/LPV (n=3), ABC/AZT/3TC/LPV (n=3), ABC/DDI/3TC/LPV (n=3), ABC/AZT/LPV (n=2), and ABC/TDF/LPV (n=1). Abbreviations: 3TC, lamivudine; ABC, abacavir; ART, antiretroviral therapy; ATV/r, atazanavir/ritonavir; LPV/r, lopinavir/ritonavir; NRTI nucleoside reverse transcriptase inhibitor; NNRTI, non-nucleoside reverse transcriptase inhibitor; PI, protease inhibitor.

### **Table S3. Characteristics of participants with a guidelines-supported treatment strategy after genotyping, by post-genotype treatment line and viral load availability, respectively.**

| **Variable** | **Total** | **Guidelines-supported post-genotype treatment strategy** | | |
| --- | --- | --- | --- | --- |
|  | **N=164 n (%)** | **Switch to**  **3^rd^ line**  **N=73 n (%)** | **Switch to different 2^nd^ line**  **N=14 n (%)** | **Stay on same**  **2^nd^ line**  **N=77 n (%)** |
| Age at genotype, median (range) | 41 (6, 70) | 41 (6, 70) | 43 (18, 68) | 41 (6, 67) |
| Female | 92 (56) | 35 (48) | 9 (64) | 48 (62) |
| Nadir CD4, median (range) | 82 (0, 1396) | 114 (0, 1396) | 66 (11, 274) | 62 (1, 935) |
| Years since nadir CD4, median (range) | 7 (0, 17) | 8 (0, 17) | 7 (2, 13) | 6 (0, 14) |
| Years on ART, median (range) | 9 (2, 17) | 10 (2, 17) | 9 (4, 13) | 8 (2, 13) |
| Years on 2^nd^-line, median (range) | 4 (0.4, 11) | 4 (1, 11) | 5 (1, 11) | 4 (0.4, 10) |
| Year of genotype, median (range) | 2017 (2011, 2021) | 2019 (2015, 2021) | 2016 (2015, 2019) | 2015 (2011, 2021) |
| ART regimen at genotype |  |  |  |  |
| TDF/3TC/ATV/r | 50 (30) | 35 (48) | 1 (7) | 14 (18) |
| TDF/3TC/LPV/r | 33 20) | 10 (14) | 6 (43) | 17 (22) |
| ABC/TDF/3TC/LPV/r | 21 (13) | 1 (1) | 3 (21) | 17 (22) |
| AZT/3TC/LPV/r | 22 (13) | 8 (11) | 3 (21) | 11 (14) |
| AZT/3TC/ATV/r | 15 (9) | 12 (16) | 0 (0) | 3 (4) |
| ABC/3TC/LPV/r | 11 (7) | 7 (10) | 0 (0) | 4 (5) |
| Other ATV/r-containing regimen^a^ | 3 (2) | 0 (0) | 1 (7) | 2 (3) |
| Other LPV/r-containing regimen^b^ | 9 (5) | 0 (0) | 0 (0) | 9 (12) |
| PI at genotype |  |  |  |  |
| ATV/r | 68 (41) | 47 (64) | 2 (14) | 19 (25) |
| LPV/r | 96 (59) | 26 (36) | 12 (86) | 58 (75) |
| Outcome post-genotype |  |  |  |  |
| VL ≤ 1000 copies/mL | 104 (63) | 62 (85) | 8 (57) | 34 (44) |
| VL > 1000 copies/mL | 41 (25) | 7 (10) | 6 (43) | 28 (36) |
| Deceased or LTFU | 6 (4) | 0 (0) | 0 (0) | 6 (8) |
| Missing^c^ | 13 (8) | 4 (5) | 0 (0) | 9 (12) |

Footnote: ^a^ Includes ABC/3TC/ATV (n=2) and ABC/TDF/3TC/ATV (n=1). ^b^ Includes AZT/TDF/3TC/LPV (n=3), ABC/AZT/3TC/LPV (n=2), ABC/DDI/3TC/LPV (n=2), and ABC/AZT/LPV (n=2). ^c^ Missing include those in care through 18 months but missing a VL within 6-18 months. Abbreviations: 3TC, lamivudine; ABC, abacavir; ART, antiretroviral therapy; ATV/r, atazanavir/ritonavir; LTFU, lost to follow-up; LPV/r, lopinavir/ritonavir; NRTI nucleoside reverse transcriptase inhibitor; NNRTI, non-nucleoside reverse transcriptase inhibitor; PI, protease inhibitor; VL, viral load.

**Table S4. Predicted resistance to the post-genotype regimen stratified by whether the post-genotype treatment strategy was in accordance with the guidelines.**

| **Discrete genotypic susceptibility score** | **Post-genotype regimen** | | |
| --- | --- | --- | --- |
|  | **Guidelines-supported 2^nd^ line**  **N=91 n (%)** | **Guidelines-supported 3^rd^ line**  **N=73 n (%)** | **Guidelines-unsupported regimen**  **N=9 n (%)** |
| 0 | 0 (0) | 1 (1) | 9 (100) |
| 1 | 14 (15) | 14 (19) | 0 (0) |
| 2 | 32 (35) | 37 (51) | 0 (0) |
| 3 | 43 (47) | 20 (27) | 0 (0) |
| 4 | 2 (2) | 1 (1) | 0 (0) |

**Table S5. Sensitivity analysis evaluating the associations between drug resistance, guidelines strategy, and VF according to hypotheses 1, 3 and 4^a^.** **Odds Ratios and 95% Confidence Intervals from G-computation.**

| **Analytic approach** | **Odds Ratios and 95% Confidence Intervals** | |
| --- | --- | --- |
|  | **G-computation** | **G-computation**  **(difference in probability of failure)** |
| Hypothesis 1 (VF by 3^rd^-line switch vs staying on 2^nd^-line) | 0.07 (0.02, 0.30) | -0.44 (-0.61, -0.21) |
| Hypothesis 3 (VF for 1 higher genotype susceptibility) | 0.80 (0.49, 1.25) | -0.05 (-0.17, 0.04) |
| Hypothesis 4 (ATV vs LPV) |  |  |
| Outcome: low or higher-level predicted DRV/r resistance |  |  |
| ATV/r vs. LPV/r | 0.94 (0.31, 3.00) | -0.01 (-0.19, 0.18) |
| Outcome: low or higher-level predicted DRV resistance |  |  |
| ATV/r only vs. LPV/r only | 0.98 (0.19, 5.92) | -0.003 (-0.20, 0.33) |
| LPV/r 🡪 ATV/r vs. LPV/r only | 0.94 (0.25, 3.09) | -0.01 (-0.17, 0.19) |
| Outcome: intermediate-high level predicted DRV/r resistance |  |  |
| ATV/r vs. LPV/r | 0.47 (0.12, 2.17) | -0.08 (-0.23, 0.08) |

### Footnote: ^a^ As only 9 participants were given a guidelines-unsupported treatment regimen, G-computation was not done for hypothesis 2. Abbreviations: ATV/r, atazanavir/ritonavir; DRV/r, darunavir/ritonavir; LPV/r, lopinavir/ritonavir; VF, viral failure.
